# The Immoral Landscape? Scientists Are Associated with Violations of Morality

**DOI:** 10.1371/journal.pone.0152798

**Published:** 2016-04-05

**Authors:** Bastiaan T. Rutjens, Steven J. Heine

**Affiliations:** 1 Department of Psychology, University of Amsterdam, Amsterdam, the Netherlands; 2 Department of Psychology, University of British Columbia, Vancouver, Canada; Tilburg University, NETHERLANDS

## Abstract

Do people think that scientists are bad people? Although surveys find that science is a highly respected profession, a growing discourse has emerged regarding how science is often judged negatively. We report ten studies (*N* = 2328) that investigated morality judgments of scientists and compared those with judgments of various control groups, including atheists. A persistent intuitive association between scientists and disturbing immoral conduct emerged for violations of the binding moral foundations, particularly when this pertained to violations of purity. However, there was no association in the context of the individualizing moral foundations related to fairness and care. Other evidence found that scientists were perceived as similar to others in their concerns with the individualizing moral foundations of fairness and care, yet as departing for all of the binding foundations of loyalty, authority, and purity. Furthermore, participants stereotyped scientists particularly as robot-like and lacking emotions, as well as valuing knowledge over morality and being potentially dangerous. The observed intuitive immorality associations are partially due to these explicit stereotypes but do not correlate with any perceived atheism. We conclude that scientists are perceived not as inherently immoral, but as capable of immoral conduct.

## Introduction

“They were mad, of course. Or evil. Or godless, amoral, arrogant, impersonal, and inhuman. They were Faust and Frankenstein, Jekyll and Moreau, Caligari and Strangelove.”

–Accompanying text to Haynes (1994) *From Faust to Strangelove*: *Representations of the scientist in western literature*.

The above quote captures a fear and distrust of scientists that may seem all too familiar. Yet these anxieties are puzzling, especially to scientists, who hold their profession in such high esteem. The results of several surveys are consistent with the notion that science is a highly respected profession [1–3]. So why would scientists be perceived in such negative terms? One reason that people might distrust scientists is that their attitudes towards science in general are often motivated by ideology. For example, when considering phenomena such as climate change [4–6], nanotechnology [7], or genetically modified food [8], people’s perceptions seem to be more influenced by whether they agree with the scientists’ conclusions.

But another reason that science may be feared is that it can seem at odds with people’s notion of morality. On the one hand, science and religion are often seen as incompatible explanatory frameworks that each aim to provide ultimate answers to the big questions in life [9–12]. The tension between science and morality is likely because religion and morality are viewed as intimately intertwined [13–15], while science often provides support for explanations at odds with religious faith. On the other hand, some have argued that science can provide the modern bedrock of morality: it shouldn’t just *describe* why people act in certain ways but should *prescribe* what is right and wrong [16–17]. The idea that both religion and science can be integral to morality has sparked much controversy and debate [14, 18–22]. Some recent research supports the parallels between science and religion in guiding moral behavior. For example, when people were primed with science-related concepts they showed greater adherence to moral norms and acted more morally, particularly pertaining to fairness and care [14, 23]. The idea behind this research is that people associate science with progress [24], which is to the benefit of everyone, and therefore offers a moral vision of society. Interestingly, these results mirror previous work reporting findings obtained with activating *religious* concepts [25–27].

However, the notion that science might offer a basis for morality is likely a minority view; for many, perhaps most, lay people, the strongest associations with morality are with religiosity. For example, recent research found an intuitive association between religious *dis*belief (i.e., atheism) and immorality [13,28]. This research utilized a classic experimental paradigm by Tversky and Kahneman [29], the conjunction fallacy or representativeness heuristic, which is based on the idea that people easily form intuitive representations of a person based on only little information. In this research [13], it was found that participants judged a variety of immoral acts (from serial murder to necrobestiality) as more representative of atheists than of various other religious, ethnic, or cultural groups, highlighting people’s perceptions that morality is built upon religious beliefs.

The current research investigates whether scientists are similarly perceived in immoral terms. Despite that scientists are among one of the more respected professions [1–3], there are a few reasons to suspect that their morality might be sometimes called into question: In addition to the potential problem that science is viewed as incompatible with religion, science may also arouse suspicions because scientific progress is frequently associated with moral decline, societal pessimism, and technological disaster [30,31]. For example, it is not uncommon to hear that the general public is anxious about the role that science plays in such feared topics as atomic energy, genetic engineering, or superbugs. Likewise, pervasive cultural archetypes of the evil and deranged scientist (e.g., Dr. Frankenstein or Dr. Strangelove, or real life examples like Josef Mengele or Ted Kaczynski) may have damaged scientists’ reputations. Moreover, there are several widely publicized cases of fraud and retractions throughout the sciences [32–34]. Yet, thus far, we know relatively little about the kinds of associations that people actually have about scientists. This is problematic because we live in a world that relies heavily on science and technology, yet in which science is also regularly critiqued and distrusted [5,6,35]. In the present research we sought to address this lacuna by testing intuitive associations between scientists and various kinds of immoral conduct, and subsequently gauging more explicit stereotypes of scientists.

### Overview of experiments

The current paper reports 10 experiments (*N* = 2328) organized around 2 sets of studies. In the first 7 studies we investigated intuitive immorality judgments of scientists, atheists, and various control targets. The final 3 studies target explicit evaluations of scientists versus other groups in an effort to shed light on people’s intuitive associations between scientists and different kinds of immoral behaviors. All research was approved by the Faculty Ethics Review Board at the University of Amsterdam (2014-SP-3818, 2014-SP-3888, 2015-SP-4027). All participants provided written informed consent before participating in the research.

## Studies 1–7

### Method

#### Participants

We approached American adults on Amazon’s Mechanical Turk and asked them to participate in a short survey on choices and values. A total of 1917 participants participated in the first 7 studies. Nine participants failed to correctly answer an *instructional manipulation check* [36] and 15 participants did not complete the study. The remaining 1893 participants were run across the 7 studies as follows: Study 1 (*N* = 266), Study 2 (*N* = 267), Study 3 (*N* = 265), Study 4 (*N* = 281), Study 5 (*N* = 281), Study 6 (*N* = 268), and Study 7 (*N* = 265). In each study, participants were randomly assigned to one of 7 conditions. The mean age was 30.04 (*SD* = 9.26, range 18–65 years), and 38% were female. Due to an oversight, in Studies 1–3 and 6–7 we did not record gender and age. See S1 Participant Demographics.

#### Procedure and materials

Participants read a description of a moral transgression committed by a man [13,37–39]. We employed a wide variety of different kinds of moral transgressions, using scenarios that have been used in past research on morality [13, 38,]). In Studies 1 and 4, the scenario depicted a man who killed 5 homeless people and buried them in his basement; Study 2’s scenario described a man who engages in consensual incest with his sister; the scenario in Studies 3 and 5 depicted a man who engages in an act of necrobestiality; Study 6 portrayed a man cheating in a card game; and Study 7 described a man ridiculing an obese woman and then kicking a dog; see S1 Scenarios). Next, participants were asked to indicate which option was more probable: A) Robert (or Jack, depending on the study) is a sports fan or B) Robert is a sports fan and {*condition*}. Depending on the condition (which was a between-groups variable), and the particular study, option B) was always one of 7 options which included—depending on the study—two or three scientist targets (*a scientist*, *a cell biologist*, *an experimental psychologist)*, an atheist target, and—depending on the study—3 or 4 of the following control targets (*a Muslim*, *Hispanic*, *a Native American*, *a Christian*, *gay*, *a psychologist*, *a teacher*, or *a lawyer*). Since it is impossible for a subset of a category to be more probable than the entire category, choosing option B indicates a reasoning error. However, as has been well-documented ever since Tversky and Kahneman’s seminal “Linda Problem” [29], people will commit the conjunction fallacy when the added target category in option B is deemed representative of the description (i.e., “active in the feminist movement”), while the original target category (i.e., “bank teller”) is not. The conjunction of both descriptions was ranked as more probable than the less representative constituent “bank teller”. In the current research, the likelihood that people will commit such an error is based on any intuitive associations between the description of the person (e.g., a serial killer) and the category (e.g., a scientist) that is selected [29].

Next, participants completed an instructional manipulation check to determine whether they were paying attention. Then, they completed demographic questions regarding their religious beliefs, ethnicity, profession, and political orientation (see S1 Participant Demographics for an overview of participant demographics across studies). Finally, participants were asked to indicate whether they believe a scientist can believe in God on a 100-point slider scale from *certainly not* (0) to *of course* (100). (This last question will be discussed under the Scientists and Religious Belief heading after Study 10).

### Results

**Fig 1:**
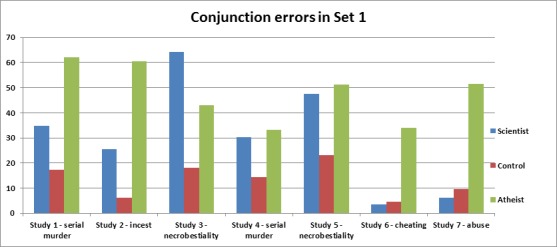
Conjunction error rates (percentages) in Studies 1–7 for each category of targets. All target groups differ at *p* < .01, except for scientist and atheist targets in Studies 4–5, and scientists and control targets in Studies 6–7.

Chi^2^ analyses were conducted in all studies to compare conjunction fallacies. In each study, we pooled the 3 scientist conditions (scientist, cell biologist, experimental psychologist), and the 4 control conditions (see S1 Conjunction Error Results for analyses with individual targets). An analysis comparing the scientist conditions with the control and atheist conditions revealed overall significant effects of target in Study 1 *χ*^2^(2) = 30.73, *p* < .001, Cramer’s *V* = .34, Study 2 *χ*^2^(2) = 46.36, *p* < .001, *V* = .42, Study 3 *χ*^2^(2) = 37.68, *p* < .001, *V* = .42, Study 4 *χ*^2^(2) = 11.90, *p* < .01, *V* = .21, Study 5 *χ*^2^(2) = 20.09, *p* < .001, *V* = .27, Study 6 *χ*^2^(2) = 40.44, *p* < .001, *V* = .39, and in Study 7 *χ*^2^(2) = 33.45, *p* < .001, *V* = .36 (see Fig 1). Subsequent analyses in Study 1 (serial murder) revealed that participants committed more errors in the scientist conditions (34.8%) than in the control conditions (17.3%; *χ*^2^(1) = 8.52, *p* < .01). Also, the atheist condition (62.0%) differed from both the scientist (*χ*^2^(1) = 10.41) and the control conditions (*χ*^2^(1) = 14.85), *p*’s < .01. In Study 2 (consensual incest), participants committed more errors in the scientist conditions (25.4%) than in the control conditions (6.1%; *χ*^2^(1) = 14.85, *p* < .001). Also, the atheist condition (60.5%) differed from both the scientist (*χ*^2^(1) = 16.34) and the control conditions (*χ*^2^(1) = 48.82), *p*’s < .001. In Study 3 (necrobestiality), participants committed more conjunction errors in the scientist conditions (64.2%) than in the control conditions (18.0%; *χ*^2^(1) = 37.75, *p* < .001). Necrobestiality was perceived as more representative of scientists than of atheists (42.9%, *χ*^2^(1) = 5.65, *p* = .019). The difference between the atheist and control conditions was also significant, *χ*^2^(1) = 9.97, *p* < .01. In Study 4 (serial murder—replication), participants committed more conjunction errors in the scientist conditions (30.4%) than in the control conditions (14.4%; *χ*^2^(1) = 8.57 *p* < .01). The scientist conditions did not differ from the atheist condition (33.3%, *p* = .74); the latter differed from the control conditions (*χ*^2^(1) = 7.99, *p* < .01). In Study 5 (necrobestiality—replication), participants committed more conjunction errors in the scientist conditions (47.4%) than in the control conditions (23.2%; *χ*^2^(1) = 14.55, *p* < .001). The scientist conditions did not differ from the atheist condition (51.3%, *p* = .70); the latter differed from the control conditions (*χ*^2^(1) = 12.20, *p* < .01). A strikingly different pattern emerged in Studies 6 and 7. In Study 6 (cheating), compared to the atheist condition (34.1%), hardly any participant committed an error in the scientist conditions (3.4%; *χ*^2^(1) = 29.95, *p* < .001). A similar difference was found when comparing the atheist condition to the control conditions (4.6%; *χ*^2^(1) = 23.27, *p* < .001). The scientist conditions did not differ from the control conditions, *p* = .74. Study 7 (abuse) revealed a similar pattern, where participants committed more errors in the atheist condition (51.4%) than in the scientist conditions (6.1%; *χ*^2^(1) = 28.04, *p* < .001) and the control conditions (9.6%; *χ*^2^(1) = 21.66, *p* < .001). Scientist and control conditions did not differ, *p* = .14.

### Discussion

These results provide a number of insights. First, replicating recent work (13), we observed an intuitive association between acts of both harmful and harmless (i.e., victimless) immorality and atheism; across all violations, atheists were more likely than controls to be intuitively associated with immorality. Second, we also found that people hold similar associations between some of the morality violations and *scientists*. Scientists were perceived as more likely than control targets to engage in disturbing violations of purity (i.e., serial murder, incest, and necrobestiality), however, they were not more likely to be perceived as being more likely to cheat or to engage in abuse. The latter finding is interesting (and encouraging) given the reasons we discussed earlier regarding how science may be distrusted for various motivational reasons. Although we had expected to find associations between scientists and purity violations, we were surprised to find no associations with fairness and care violations. This pattern of results can be interpreted in the light of Moral Foundations Theory (MFT; [40,41]). MFT maintains that there are two broad classes of moral foundations: Binding foundations are those intuitions that bind people into roles and duties as a means to allow them to live harmoniously with others. In contrast, individualizing foundations are those intuitions that emphasize suppressing selfishness and learning to respect the rights of others. Cheating/fairness and care/abuse (harm) reflect the two individualizing moral foundations. In contrast, the scenarios depicting serial murder, incest, and necrobestiality are primarily examples of purity/degradation, which is one of the 3 binding foundations (together with authority/subversion and loyalty/betrayal; perhaps a willful disrespect of the law as depicted in all the scenarios employed also reflects the authority/subversion foundation). Moreover, serial murder is obviously also an *extreme* example of harm. We will turn to Moral Foundations Theory in the next study to substantiate our findings and examine this interpretation in more detail.

## Studies 8–10

What is it about scientists that triggers the observed intuitive association with immorality? That there was no association with cheating and abuse but only with more extreme purity transgressions, might suggest that there is some truth to the stereotype of a scrupulous “Faustian experimentalist” unburdened by morality but not deliberately evil. To further explore people’s perceptions of scientists’ morality, we conducted a final set of studies in which we assess explicit evaluations of scientists. Study 8 assesses moral stereotypes [42], and Studies 9–10 investigate more general stereotypes and perceived preferences, values, and motivations. Moreover, we explore how people’s views of scientists relate to their conjunction fallacies in Studies 9 and 10.

## Study 8

Thus far we have found that scientists are intuitively associated with a range of disturbing norm violations and acts of immorality, however, they were not associated with comparatively mild violations pertaining to fairness and care. In Study 8, we aimed to assess moral stereotypes of scientists by more directly exploring the moral foundations that are associated with them. Given the observed pattern of results, we expected that scientists would be associated with a lower endorsement of the purity/degradation foundation. At the same time, we expected no differences in endorsement of the care/harm and fairness/cheating foundations.

### Method

One hundred and thirteen American adults (35% female, mean age = 31.13, SD = 9.57) from Amazon’s MTurk participated in the study. Participants completed the moral judgment section of the Moral Foundations Questionnaire (MFQ30-part2; [40]) but from the perspective of “John,” who was described as either a scientist or a sports fan (42). There were 15 items and 1 control item (“It is better to do good than to do bad”) which cover the 5 moral foundations of care/harm, fairness/cheating, loyalty/betrayal, authority/subversion, and purity/degradation. Participants were instructed as follows: “The following questions are about John. John is a sports fan (or scientist; depending on condition). What we would like you to do is respond to the items below like you believe *John would respond*. Of course you do not know John in person, but please try to respond as John would to the best of your ability.” Scores on all 16 items ranged from 1 (*John strongly disagrees*) to 5 (*John strongly agrees*). Example items are “Compassion for those who are suffering is the most crucial virtue” (care/harm), “Justice is the most important requirement for a society” (fairness/cheating), “It is more important to be a team player than to express oneself” (loyalty/betrayal), “Respect for authority is something all children need to learn” (authority/subversion), “People should not do things that are disgusting, even if no one is harmed” (purity/degradation). Alphas were .53 (Harm), .44 (Fairness), .54 (Ingroup), .70 (Authority), and .54 (Purity), which reflects previous work [40]. Note that each subscale consisted of only 3 items.

After completing the moral foundation questions for John, participants answered the same set of items in the first person. Then, immediately afterwards, participants were presented with a manipulation check item asking them to describe what they remember about John. Twelve out of 110 participants were not able to correctly report that John was either a scientist or a sports fan and were therefore excluded from the analyses, as were 3 participants who did not complete the study. Participants then completed an instructional manipulation check, demographics, and indicated whether they think a scientist can believe in God. We also added the slightly different question “Compared to a regular person, how much do you think that the average scientist believes in God?”. (We use the term “regular person” as a shorthand to mean a non-scientist, and by no means are implying that scientists are not regular people as well. Note that responses to the slightly differently worded “How much do you think that a scientist typically believes in God?” were very similar). The results can be found in the ‘Scientists and Religious Belief’ section.

### Results and Discussion

We averaged the three responses for each moral foundation, thus creating five indices on which higher scores reflect greater endorsement of that particular foundation (see Table A in S1 File, which include test statistics and effect sizes).

**Fig 2:**
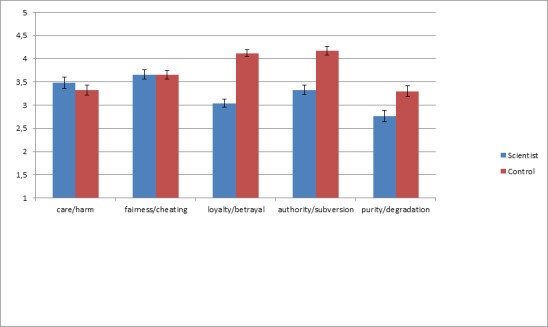
Perceived endorsement of moral foundations by scientist versus control target, scored on 5-point scales ranging from 1 (*strongly disagrees*) to 5 (*strongly agrees*), controlling for perceived atheism of scientists. Differences were significant for loyalty/betrayal, authority/subversion, and purity/degradation (all *ps* < .01). Error bars show standard errors of the mean.

Fig 2 shows that scientists are overall perceived as less likely than a control group to endorse the binding moral foundations: loyalty/betrayal (*F*(1, 97) = 84.62, *p* < .001, η^2^_p_ = .47), authority/subversion (*F*(1, 97) = 30.02, *p* < .001, η^2^_p_ = .24), and purity/degradation (*F*(1, 97) = 9.17, *p* < .01, η^2^_p_ = .09). Although our previous studies led us to expect such a pattern for purity/degradation, we had not predicted any differences for the other two binding dimensions; strikingly, these were the foundations which yielded the strongest effects. Consistent with Studies 6–7, there were no differences for the individualizing foundations of care/harm and fairness/cheating. These results were not impacted by covarying participants’ own moral foundation scores or political orientation.

The scenarios depicting violations of fairness and care that we employed in Studies 6–7 might be interpreted as less ‘extreme’ or ‘weird’ than the scenarios depicting violations of the binding moral foundations (Studies 1–5). Study 8 addressed this issue by employing the Moral Foundations Questionnaire instead of scenarios (and corroborating the results obtained in Studies 1–7).

In short, scientists are morally stereotyped as more degraded, more subversive, and less loyal than the sports fan control group. However, they are not seen as any different in their motivations for care and fairness.

## Study 9

### Method

One hundred and sixteen American adults from Amazon’s MTurk were asked to participate in a short survey. Five participants were excluded because of incomplete responses. The mean age of the remaining 111 participants (38% female) was 32.56 (SD = 11.34). Participants were first presented with a conjunction fallacy task. They read a short description of a person eating their deceased pet dog (a purity violation; 38) and were asked whether the person was a sports fan or a sports fan and a scientist. Then, they rated 5 groups on 14 Likert scales; a scientist, an experimental psychologist, an atheist, a regular person, and a lawyer. For each target group, participants were instructed to indicate the extent to which these traits generally applied (order was randomized), using slider scales ranging from 0 (*totally disagree*) to 100 (*totally agree*). The traits reflected various stereotypes and were primarily based on the Stereotype Content Model [43] and Moral Foundations Theory [40]; items can be found in Table 1). After rating all groups, participants completed an instructional manipulation check, demographic measures, and 2 questions about scientists’ religiosity.

**Table 1:** Stereotypes of scientist, experimental psychologist, atheist, lawyer, and regular person targets in Study 9. All scales ranged from 1 (*totally disagree*) to 100 (*totally agree*). SD’s in parentheses.

	Scientist	Experimental Psychologist	Atheist	Lawyer	Regular Person
*Scrupulous*	58.06 (25.24)a	54.99 (22.18)ab	52.23 (21.68)ab	48.05 (25.81)b	49.97 (17.58)b
*Nerdy*	81.16 (17.25)a	66.23 (20.04)b	44.85 (23.95)c	42.74 (23.79)c	49.97 (17.58)c
*Like a robot*	43.77 (26.19)a	40.41 (25.63)a	28.41 (22.09)b	48.40 (28.22)a	27.98 (21.70)b
*Happy*	57.32 (19.06)ab	52.42 (19.54)a	60.38 (22.22)b	44.33 (21.10)c	58.41 (15.08)b
*Imperturbable*	48.25 (22.42)ab	50.73 (20.68)a	44.06 (21.50)ab	51.48 (21.23)a	41.17 (17.60)b
*Goal-oriented*	84.22 (17.05)a	77.64 (19.68)b	56.27 (22.23)c	81.78 (18.28)ab	58.57 (17.06)c
*Lacks emotions*	43.23 (23.99)a	41.76 (25.87)a	29.29 (24.70)b	55.09 (25.96)c	26.90 (19.40)b
*Cold*	45.95 (24.17)a	42.57 (23.54)a	37.95 (25.42)ab	63.77 (23.51)c	32.90 (18.81)b
*A cheat*	16.88 (15.95)a	24.25 (20.63)b	25.02 (21.79)b	54.93 (25.38)c	34.23 (19.98)d
*Subversive*	37.47 (22.80)ab	36.23 (21.46)a	41.42 (23.47)ab	44.82 (25.80)b	38.18 (20.48)ab
*Trustworthy*	71.72 (17.25)a	55.59 (22.40)b	62.16 (22.88)b	38.60 (23.89)c	56.99 (19.39)b
*Loves his country*	49.30 (19.99)a	49.95 (19.92)a	51.01 (22.13)a	49.69 (20.63)a	62.78 (16.89)b
*Disobedient*	27.08 (21.24)a	27.41 (20.02)a	43.47 (24.25)b	35.45 (23.48)bc	35.02 (19.16)c
*Liberal*	62.14 (22.50)a	61.68 (18.08)a	70.54 (25.51)b	49.30 (20.89)c	49.68 (12.36)c

*Note*. Means within rows with different letters (a, b, c)differ significantly at the .05 threshold; all comparisons were adjusted for multiple comparisons using Bonferroni correction.

### Results

Of the 111 participants, 42 (37.8%) committed the conjunction fallacy.

Table 1 shows that scientists are perceived as significantly more nerdy, robot-like, goal-oriented, and emotionless than regular persons and atheists. Scientists were also perceived as more scrupulous, cold, liberal, and less loving of their country than regular persons but these evaluations of scientists did not significantly differ from the atheist evaluations.

Next, we assessed whether the stereotypical evaluations of scientists would be statistically related to the conjunction fallacy outcomes. We found that the only evaluations that correlated (very modestly) with the conjunction fallacy result were whether people perceived scientists to be ‘like a robot’ (*r* = .19, *p* = .044) or to ‘lack emotions’ (*r* = .18, *p* = .057). Put differently, the participants who viewed scientists as being more likely to eat their deceased dog scored somewhat higher on these two evaluative scales of scientists (see Table B in S1 File for all correlations). However, controlling for fallacy outcome did not meaningfully affect any of the reported differences in stereotypes between the different target groups that are presented in Table 1.

Interestingly, explicit evaluations of *lawyers* were in many cases similar to those of scientists, and sometimes even more extreme, with the exception that scientists were seen as more scrupulous, nerdy, and liberal than lawyers. However, in Studies 4–5, we utilized a lawyer target and did not observe a strong intuitive association with two different moral violations (serial murder and necrobestiality). Thus, while lawyers are explicitly evaluated to be quite similar to scientists on a number of the stereotype scales utilized in Study 9, they were not implicitly associated with morality violations.

### Discussion

Scientists were seen as less disobedient, and more trustworthy and less “a cheat” than the other groups, although the latter was less the case for experimental psychologists. These results are interesting because they indicate that people do not rate scientists as disobedient or as dishonest. This is consistent with the results of Studies 6–7 and resonates with the generally high levels of respect and prestige that scientists are seen to possess [1–3]. Rather, people seem to stereotype scientists as somewhat *inhuman*, rating them as robot-like and emotionless [2], goal-oriented, and to some extent scrupulous. This might suggest that it is not so much the case that people view scientists as immoral villains, but rather that they believe that scientists might be driven by other motivations that can sometimes supersede the motivation to act morally or abide to social norms. In a final study, we more extensively investigate some of these other motivations.

## Study 10

### Method

**Fig 3:**
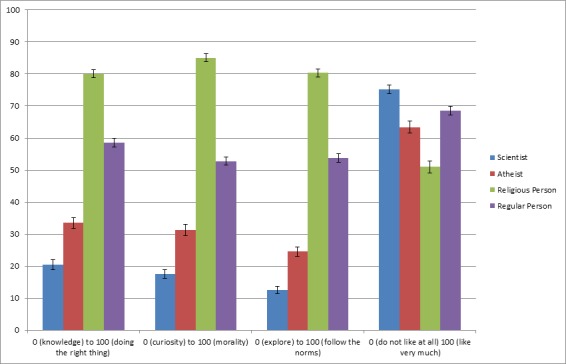
Evaluations of preferences and values, and likability, of the target groups (Study 10). Within items, all means differ significantly from each other at *p* < .01 (Bonferroni adjusted); the only non-significant difference was liking of atheists versus regular persons). The third item was presented to participants from 0 (*follow the norms*) to 100 (*explore*), but reverse-scored in the current Fig. Error bars show standard errors of the mean.

**Fig 4:**
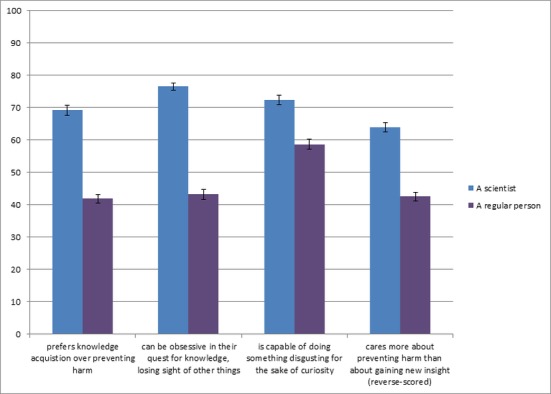
Evaluations of motivational trade-offs of scientists and control targets, Study 10. All means differ at *p* < .001. Error bars show standard errors of the mean.

Two hundred and twenty-six American adults (48% female; average age = 35.93, SD = 12.22) from Amazon’s MTurk participated. The study consisted of three short parts. First, participants were presented with the same morality violation description as in Studies 3 and 5 (necrobestiality) and were asked whether the target was a sports fan, or a sports fan and a scientist. Next, participants evaluated four target categories (scientist, atheist, religious person, regular person) on 100-point slider scales in terms of “What do the following groups of people prefer or value more?” The endpoints of the scale are presented in Fig 3. To investigate potential halo effects, participants also indicated how much they liked each category of people. Next, they indicated their agreement on a 100-point scale with four statements about scientists versus regular persons (see Fig 4 for the items). All items were designed to tap into a perceived motivational trade-off and investigated the extent participants believe that scientists value–-and are driven primarily by—exploration and knowledge gain, in contrast to morality concerns. Participants then rated on 100-point unipolar slider scales how much they thought scientists, atheists, and ordinary persons are ‘mad’, ‘bad’, and ‘dangerous’ [44]. Finally, participants completed the same demographic questions as in previous studies, and 2 questions about scientists’ beliefs in God. Detailed analyses can be found in S1 File.

### Results

Overall, 81 participants (36% of the sample) committed the conjunction fallacy with the scientist target.

**Fig 5:**
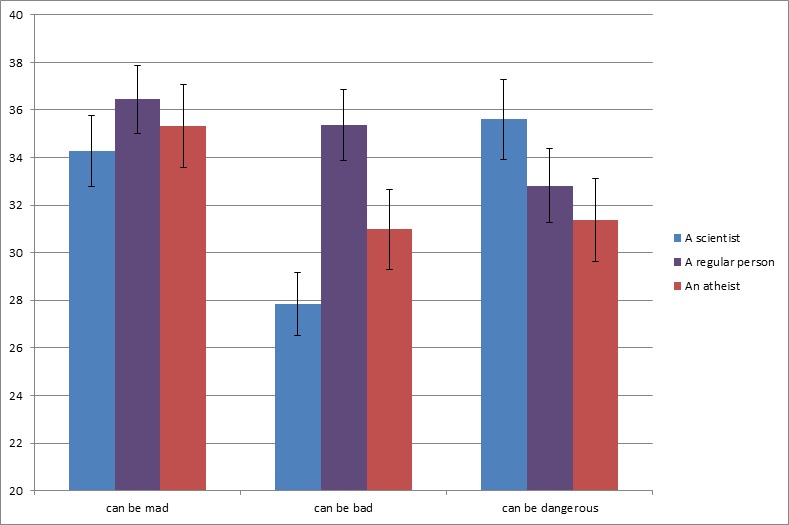
Stereotype measure, Study 10. Whereas ‘Can be mad’ stereotype did not significantly vary across groups, all ‘Can be bad’ means differ significantly at *p* < .05. ‘Can be dangerous’ was higher for scientist target than for regular person target (*p* = .053) and atheist target (*p* < .05). Error bars show standard errors of the mean.

As can be seen in Fig 3, scientists are evaluated as preferring and valuing knowledge, curiosity, and exploration over doing the right thing, morality, and following the norms, more so than any of the control groups (all *p*’s < .001). At the same time, scientists are the most liked group (all comparisons at *p* < .01), which renders a halo effect unlikely in accounting for the conjunction fallacy in this, and previous studies. Moreover, Fig 4 shows that scientists are perceived as being more motivated than the average person to acquire knowledge, satisfy their curiosity, and gain new insights, at the expense of the prevention of harmful or disgusting consequences of their actions (all *p*’s < .001). Fig 5 shows that scientists are not perceived as potentially more mad than the other targets, and that they are viewed as potentially less bad than the other targets (both *p*’s < .05). However, they are perceived as potentially more dangerous than atheists and regular persons (both *p*’s < .05). A more detailed description of these analyses can be found in S1 File.

Next, we again assessed which of the evaluations of scientists were statistically related to the conjunction fallacy outcomes (see Table C in S1 File for all correlations). In addition to the correlations in Study 9, such a relation would shed some light on what might drive the *intuitive* association of scientists with immoral conduct. Intuitively judging a morality violation (necrobestiality) as representative of scientists correlated modestly with evaluations of scientists valuing knowledge (*r* = .15) and curiosity (*r* = .15) over “doing the right thing” and morality. The fallacy measure also has modest positive correlations with the potentially mad (*r* = .18), bad (*r* = .18), and dangerous (*r* = .21) stereotypes; interestingly, the latter also reflects the one stereotype that was more strongly endorsed for scientists than for the other targets. Interestingly, controlling for fallacy did not alter the above results except for the “Can be dangerous” stereotype. Here, we observe that the effect of target category on the ‘dangerous’ ratings was only significant for participants who committed the fallacy (scientist associated with necrobestiality). In other words, there is a relation between associating scientists with immoral behavior and perceiving them as potentially dangerous. All correlations remained significant (all *p*’s < .05) when controlling for liking. There were no significant correlations between valuing norms over exploration or for liking of scientists (both *r*s < .04).

While Study 9 found that intuitively associating immorality violations with scientists modestly correlated with stereotypes of scientists as an emotionless robot-like person, the current study found that immorality violations correlated with the perception of scientists as primarily valuing knowledge and curiosity, as well as with seeing scientists as potentially mad, bad, and dangerous.

### Discussion

Study 10 shows that scientists’ preferences, values, and motivations are perceived as different from the motivations of other groups of people, including atheists. Indeed, scientists are perceived as preferring and valuing knowledge and exploration over maintaining morality, more so than any of the comparison targets. At the same time, this does not appear to lead people to dislike scientists or to evaluate them as bad or mad; participants only viewed scientists as potentially more *dangerous* compared to atheists and regular people.

Together, the results of Studies 9 and 10 suggest that people view scientists as goal-oriented, emotionless robots who favor knowledge and exploration over maintaining purity and social norms. In the public eye, scientists thus seem to exist more in an *amoral* landscape than in an immoral one.

## Scientists and Religious Belief

### Method

Across all studies, participants were asked to respond to the following question: “Do you think that a scientist can believe in God?” In studies 8–10 we added two differently worded items. They also indicated whether they themselves work in academia or are scientists (9.5% were).

### Results

Participants overall seem to agree that scientists can believe in God, although they believe that scientists are somewhat less likely than a regular person to do so (see Table 2). We also assessed whether these responses correlated with the conjunction fallacy rates for scientist targets by combining the data of studies 1–5 and 9–10. The overall correlation of fallacy rates with “Can a scientist belief in God’ was *r* = -.003, *p* = .94 (*N* = 543); for both of the other two items correlations were *r* < .07 (*p’s* >.35). Participants’ self-reported belief in God did also not correlate with the fallacy (*r* = .06, *p* = .15), nor did their political orientation (*r* = .07, *p* = .12). Intuitive associations of scientists with immoral conduct were therefore not likely due to any perceived atheism.

**Table 2:** Responses to scientists and religious belief items, and occupation of respondents, across studies. SD’s in parentheses. All items scored on scales ranging from 0 (*not at all)* to 100 (*very much so*).

	*Studies 1–7*	*Study 8*	*Study 9*	*Study 10*
“*Can a scientist believe in God*?*”*	70.19 (33.48)	71.83 (29.86)	65.71 (33.52)	77.15 (28.40)
“*Compared to a regular person*, *how much do you think that the average scientist believes in God*?”		32.58 (18.44)		36.17 (21.29)
“*How much do you think that a scientist typically believes in God*?*”*			34.95 (21.12)	

## General Discussion

The current work is the first to systematically investigate morality judgments of scientists, which is highly relevant given that we live in a world that is heavily invested in science and technology. Taken together, the results of 10 studies shed light on how the general public perceives scientists in terms of their morality: overall, scientists provoke decidedly mixed associations. While scientists are largely trusted (and liked), they are also viewed as somewhat inhuman and obsessed enough with the pursuit of knowledge that they are perceived as capable of immoral conduct and can be potentially dangerous. Indeed, people’s intuitive associations of scientists are unusual enough that they view them as a good fit for a host of highly disturbing behaviors. In general, the frightening behaviors associated with scientists were more likely violations of binding rather than individualizing moral foundations, particularly purity violations. These associations were observed for the general target category of ‘scientists’ as well as for the specific target categories of ‘cell biologists’, and ‘experimental psychologists’. Although it is possible that the specific target categories represent the general scientist category better than, say, a physicist or a political scientist, we do not believe this is likely; using targets from two different domains of science as well as the general category led to similar associations and evaluations across Studies 1–7 and 9.

These associations and stereotypes about scientists provide additional insight into the ideological rejection and distrust of science and scientific findings that many display [5,6,35].

This research also informs discussions regarding the link between religion and morality [13,14,18,22]. First, we provide a comprehensive replication of Gervais [13]. However, while religion might be intuitively viewed by many as the cornerstone of morality, the current experiments show that atheists are not alone in the immoral landscape. Serial murder, incest, and necrobestiality were judged as representative of both scientists and atheists, whereas cheating and abuse were seen as representative of atheists but not of scientists. These results resonate with research discussed earlier which showed that priming science-concepts increases adherence to fairness and care norms [14,23].

However, the current results do not seem to be due to scientists being simply perceived as a subcategory of atheists. Across studies scientists were not seen as unlikely to believe in God, and people’s judgments about scientists’ religiosity did not predict their conjunction fallacies. Moreover, atheists were perceived to act unfairly and to commit abuse, unlike scientists. In addition, we found that people held quite different stereotypes and evaluations about scientists compared with atheists. Scientists and atheists appear to be perceived quite differently.

Trustworthiness is an obvious variable for immorality perceptions [28], yet scientists were rated as more trustworthy than the other targets in our studies, which is consistent with scientists being among the most respected professions [1–3]. This result is interesting and encouraging, given the well-documented distrust of science on politically loaded topics (e.g., climate change, GMOs) as well as the aftermath of recent highly publicized fraud cases in academia. Rather than untrustworthy, it seems that scientists are viewed as somewhat unpredictable, in that they can be potentially dangerous and commit severe acts of immoral conduct. They are not perceived this way because they are seen as evil but more likely because they are seen—as shown in Study 10—to pursue knowledge obsessively and in the process might lose sight of what is moral. It is therefore not surprising that people can easily believe that scientists commit disturbing morality violations, possibly as a side-effect of their curiosity and search for knowledge.

### Limitations

The unusual intuitive associations that people have with the category of scientist were all assessed using the conjunction fallacy. It is possible that this measure does not necessarily tap into people’s representations of the *prototypes* of categories, but instead captures people’s representations of the full *breadth* of these categories. The scenarios in Studies 1–5 depicted decidedly extreme and disturbing behavior, so participants might have been searching for a category that was broad and unusual enough to include individuals that could act in such strange and alarming ways. The category of scientist might be seen as representative of these scenarios, even if it is perceived as largely consisting of respectable and morally upright individuals (e.g., Marie Curie, Albert Einstein, Gregor Mendel), because the category may also be seen as containing a distinct lunatic fringe. That is, the boundaries of people’s category of scientists might stretch further out into the immoral landscape than they do for the control categories that we employed.

Related to the notion of boundaries discussed above, it is interesting that—as can be seen in S1 Conjunction Error Results—Christian targets in Studies 1 and 4 (but not the other control targets) were also associated with serial murder to a relatively strong degree. This was however not the case for the incest and necrobestiality scenarios. We did not predict this effect beforehand, but it is possible that the ‘Christian’ category is broad enough that it can contain both moral as well as some forms of extremely immoral behavior (e.g., serial killers explicitly inspired by religion or some particular Christian symbolism).

It is worth noting that people assumed that scientists were more likely to violate the binding moral foundations, which are the same moral foundations that conservatives tend to embrace more than liberals [40]. Perhaps, then, our participants were just assuming that scientists are liberal, and responded as such. However, we included other control targets that also could be deemed liberal: for example, psychologist, gay, and teacher, yet none of these showed the same pattern of alignment with (violations of) the binding foundations that people assumed for scientists. Moreover, respondents reported viewing scientists as less liberal than they did atheists. Thus, we do not think that merely viewing scientists as liberals provides a plausible alternative account for our findings.

These studies relied exclusively on samples of American MTurk workers and it remains an open question whether the results would generalize (but see [45], on the diversity and representativeness of MTurk samples). On average, MTurk workers tend to be better educated and more liberal [46] and thus more similar to scientists [47] than other Americans. If anything, a more representative sample of Americans should have even more negative views of scientists. Moreover, given that attitudes towards science vary around the world [48,49], we anticipate that people’s intuitive associations with science would also vary accordingly.

It is notable that all the scenarios in our studies involved male protagonists, so perhaps people only associate disturbing behaviors with *male scientists*. It would be informative to see what behaviors are associated with female scientists, but a problem with addressing this is that people’s implicit associations with scientists are decidedly male [50].

### Conclusion

Taken together, the current work shows that people perceive scientists not as unequivocally bad, but rather their perceptions are a complex mixture of positive and negative stereotypes and associations. The results thus suggest that scientists are seen as existing within more of an amoral, as opposed to an immoral, landscape in which immoral consequences might be preceded by amoral causes.

## Supporting Information

S1 Conjunction error results (DOCX)

S1 File (DOCX)

S1 Participant demographics (DOCX)

S1 Scenarios (DOCX)
