# Supplementary material for: The Immoral Landscape? Scientists Are Associated with Violations of Morality
Source: PLoS One. 2016 Apr 5;11(4):e0152798. doi: 10.1371/journal.pone.0152798 (PMC4821584; doi:10.1371/journal.pone.0152798)
Supplement: S1 Conjunction error results — (DOCX) [file pone.0152798.s001.docx]

**S3 Conjunction error results**

Table 1.

Proportion of committed conjunction errors in Study 1.

| ***Study 1 – Serial killer scenario*** | **Fallacy** | **N** |
| --- | --- | --- |
| Scientist | 27.8% | 36 |
| Cell biologist | 32.4% | 34 |
| Experimental psychologist | 42.9% | 42 |
| Atheist | 62.0% | 50 |
| Muslim | 5.6% | 36 |
| Christian | 32.4% | 34 |
| Gay | 14.7% | 34 |

Table 2.

Proportion of committed conjunction errors in Study 2.

| ***Study 2 – Incest scenario*** | **Fallacy** | **N** |
| --- | --- | --- |
| Scientist | 24.5% | 49 |
| Cell biologist | 10.0% | 40 |
| Experimental psychologist | 41.5% | 41 |
| Atheist | 60.5% | 38 |
| Muslim | 5.0% | 40 |
| Christian | 12.0% | 25 |
| Gay | 2.9% | 34 |

Table 3.

Proportion of committed conjunction errors in Study 3.

| ***Study 3 – Necrobestiality scenario*** | | **Fallacy** | | **N** |
| --- | --- | --- | --- | --- |
| Scientist | | 62.8% | | 43 |
| Experimental Psychologist | | 65.8% | | 38 |
| Psychologist | | 21,7% | | 46 |
| Atheist | | 42.9% | | 49 |
| Native American | | 7.1% | | 28 |
| Christian | | 20.7% | | 29 |
| Gay | | 25.0% | | 32 |
| Table 4. Proportion of committed conjunction errors in Study 4. | |  | |  |
| ***Study 4 – Serial killer scenario*** | **Fallacy** | | **N** |  |
| Scientist | 27.5% | | 40 |  |
| Experimental Psychologist | 33.3% | | 39 |  |
| Atheist | 33.3% | | 42 |  |
| Teacher | 12.5% | | 40 |  |
| Lawyer | 12.2% | | 41 |  |
| Muslim | 5.1% | | 39 |  |
| Christian | 27.5% | | 40 |  |

Table 5.

Proportion of committed conjunction errors in Study 5.

| ***Study 5 – Necrobestiality scenario*** | **Fallacy** | **N** |
| --- | --- | --- |
| Scientist | 46.2% | 39 |
| Experimental Psychologist | 48.7% | 39 |
| Atheist | 51.3% | 39 |
| Teacher | 24.4% | 41 |
| Lawyer | 35.7% | 42 |
| Native American | 7.1% | 42 |
| Christian | 25.6% | 39 |

Table 6.

Proportion of committed conjunction errors in Study 6.

| ***Study 6 – Fairness violation scenario*** | **Fallacy** | **N** |
| --- | --- | --- |
| Scientist | 0.0% | 37 |
| Cell biologist | 2.4% | 42 |
| Experimental psychologist | 7,5% | 40 |
| Atheist | 34.1% | 41 |
| Muslim | 5.0% | 40 |
| Christian | 8.8% | 34 |
| Gay | 0.0% | 34 |

Table 7.

Proportion of committed conjunction errors in Study 7.

| ***Study 7 – Care violation scenario*** | **Fallacy** | **N** |
| --- | --- | --- |
| Scientist | 0.0% | 30 |
| Experimental Psychologist | 12.2% | 49 |
| Atheist | 51.4% | 35 |
| Psychologist | 2.6% | 38 |
| Hispanic | 5.0% | 38 |
| Christian | 23.5% | 34 |
| Gay | 7.3% | 41 |
