# Supplementary material for: The Immoral Landscape? Scientists Are Associated with Violations of Morality
Source: PLoS One. 2016 Apr 5;11(4):e0152798. doi: 10.1371/journal.pone.0152798 (PMC4821584; doi:10.1371/journal.pone.0152798)
Supplement: S1 File — (DOCX) [file pone.0152798.s002.docx]

**S1 File**

Table A. Perceived endorsement of moral foundations by scientist versus control target in Study 8. SE’s in parentheses. All results are Bonferroni-corrected, and controlled for perceived atheism of scientists. Further controlling for political orientation and participants’ own moral foundation scores did not meaningfully alter these results.

|  | **Scientist**  ***N* = 46** | **Control (Sports fan)**  ***N* = 52** | **Test statistics** |
| --- | --- | --- | --- |
| care/harm | 3.48 (.90) | 3.32 (.67) | *F*(1, 97) = .93, *p* = .34, η^2^_p_ = .01 |
| fairness/cheating | 3.66 (.68) | 3.65 (.62) | *F*(1, 97) = .01, *p* = .93, η^2^_p_ = .00 |
| loyalty/betrayal | 3.04 (.57) | 4.12 (.55) | *F*(1, 97) = 84.62, *p* < .001, η^2^_p_ = .47 |
| authority/subversion | 3.33 (.79) | 4.17 (.67) | *F*(1, 97) = 30.02, *p* < .001, η^2^_p_ = .24 |
| purity/degradation | 2.76 (.90) | 3.30 (.72) | *F*(1, 97) = 9.17, *p* < .01, η^2^_p_ = .09 |
| control item | 4.59 (.72) | 4.42 (.61) | *F*(1, 97) = 1.85, *p* = .18, η^2^_p_ = .02 |

Table B. Correlations of conjunction fallacy and explicit evaluations in Study 9. **p* < .05, ^*p* < .06

|  | *Fallacy* |
| --- | --- |
| *Scrupulous* | -.11 |
| *Nerdy* | .06 |
| *Robot* | .19* |
| *Happy* | .08 |
| *Imperturbable* | -.02 |
| *Goal-oriented* | -.09 |
| *Lacking emotions* | .18^ |
| *Cold* | -.04 |
| *A cheat* | .07 |
| *Subversive* | .15 |
| *Trustworthy* | -.07 |
| *Loves country* | .16 |
| *Disobedient* | .05 |
| *Liberal* | -.15 |

Table C. Correlations of conjunction fallacy and explicit evaluations in Study 10. **p* < .05

|  | *Fallacy* |
| --- | --- |
| *knowledge* over *right thing* | -.15* |
| *curiosity* over *morality* | -.15* |
| *norms* over e*xploration* | .03 |
| *liking* | -.04 |
| *knowledge over harm* | .04 |
| *obsessive in quest for knowledge* | .11 |
| *capable of doing something disgusting* | .09 |
| *cares about preventing harm* | -.03 |
| *mad* | .18* |
| *bad* | .18* |
| *dangerous* | .21* |

Table D. Evaluations of preferences and values, and likability, of the target groups, Study 10. SD’s in parentheses. Within-subject test statistics are Huynh-Feldt corrected (Greenhouse-Geisser corrected for *liking*). All means within all rows differ significantly at *p* <.001 (Bonferroni adjusted), except for *liking* (scientists were liked more than the other groups [all *p*’s <.01], the only non-significant difference was for atheists versus regular persons). The third item was presented to participants from 0 (*follow the norms*) to 100 (*explore*), but reverse-scored in the current table.

| ***What do the following groups prefer or value?*** | | | | | |
| --- | --- | --- | --- | --- | --- |
| *Scale* | ***Scientist*** | *Atheist* | *Religious person* | *Regular person* | ***Test statistics*** |
| 0 (*knowledge*) to 100 (*doing the right thing*) | **20.47** ***(22.93)*** | 33.52  (24.90) | 79.99  *(18.80)* | 58.54  *(19.85)* | *F =* 353.32*, p*<.001*,* η^2^_p_ = .61 |
| 0 (*curiosity*) to 100 (*morality*) | **17.54**  ***(20.16)*** | 31.24  *(27.09)* | 84.91  *(17.07)* | 52.82  *(17.50)* | *F =* 440*.14, p*<.001*,* η^2^_p_ = .66 |
| 0 (*explore*) to 100 (*follow the norms*) | **12.58**  ***(16.78)*** | 24.54  *(20.73)* | 80.50  *(22.11)* | 53.79  *(19.25)* | *F =* 500.23*, p*<.001*,* η^2^_p_ = .69 |
| ***How much do you like people that belong to the following groups?*** | | | | | |
|  | ***Scientist*** | *Atheist* | *Religious person* | *Regular person* | ***Test statistics*** |
| 0 (*do not like at all*) 100 (*like very much*) | **75.19**  **(21.78)** | 63.41 (29.06) | 50.96  (29.19) | 68.51  (21.29) | *F =* 39.74, *p*<.001*,* = .15  |

Table E. Evaluations of motivations of scientists and control targets and stereotype measure, Study 10. SD’s in parentheses. Motivations were only measured for scientist and regular person targets. There were some missing values on the stereotypes measures (see DF’s). Within rows of the stereotype measure, means with different subscripts differ significantly (LSD comparisons) at *p* < .05, except ¥ = *p* =.053

|  | ***A scientist…*** | *A regular person…* | *An atheist…* | ***Test statistics*** |
| --- | --- | --- | --- | --- |
| **Motivations evaluations from 0 (*Disagree*) to 100 (*Agree*)** | | | |  |
| *…prefers knowledge acquisition over preventing harm* | **69.24 (22.75)** | 41.88  (20.86) | - | *F*(1, 225) = 212.66, *p* <.001, η^2^_p_ = .49 |
| *…can be obsessive in their quest for knowledge, losing sight of other things* | **76.54**  **(17.67)** | 43.22  (22.75) | - | *F*(1, 225) = 333.16, *p* <.001, η^2^_p_ = .60 |
| *…is capable of doing something disgusting for the sake of curiosity* | **72.35**  **(21.06)** | 58.72  (23.17) | - | *F*(1, 225) = 49.66, *p* <.001, η^2^_p_ = .18 |
| *…cares more about preventing harm than about gaining new insights* (reverse-scored) | **64.03**  **(21.88)** | 42.51  (19.92) | - | *F*(1, 225) = 136.50, *p* <.001, η^2^_p_ = .38 |
| **Stereotype: *To what extent do you think that…*** | | | |  |
| *…can be mad* | **34.27^a^**  **(22.14)** | 36.44^a^  (21.45) | 35.32^a^  (26.06) | *F*(2, 444) = 1.08, *p* = .34, η^2^_p_ = .01 |
| *…can be bad* | **27.85^a^**  **(19.71)** | 35.35^b^  (22.16) | 30.99^c^  (25.04) | *F*(2, 442) = 15.72, *p* < .001, η^2^_p_ = .07 |
| *…can be dangerous* | **35.60^a^**  **(25.14)** | 32.82^b¥^  (23.13) | 31.36^b^  (25.89) | *F*(2, 440) = 4.26, *p* = .015, η^2^_p_ = .02 |
