# Supplementary material for: The Immoral Landscape? Scientists Are Associated with Violations of Morality
Source: PLoS One. 2016 Apr 5;11(4):e0152798. doi: 10.1371/journal.pone.0152798 (PMC4821584; doi:10.1371/journal.pone.0152798)
Supplement: S1 Participant demographics — (DOCX) [file pone.0152798.s003.docx]

**S1 Participant demographics**

Table 13. Overview of participant demographics, across studies.

| Total *N* | 2328 |
| --- | --- |
| Data collection period | August 2014 – January 2015 |
| Mean Age* | 32.42 (*SD* = 10.60) |
| Gender* | 61% male, 39% female |
| Religious: yes or no? | Yes: 658 (28.3%)  No: 1670 (71.7%) |
| Reported religious affiliation | Protestant: 350 (15.0%)  Catholic: 280 (12.0%)  Jewish: 47 (2.0%)  Buddhist: 22 (0.9%)  Agnostic: 327 (14.0%)  Atheist: 410 (17.6%)  None: 668 (28.7%)  Other: 224 (9.6%) |
| ‘Do you believe in God or a higher power?’  *not at all* (0) to *very much* (100) | 40.18 (*SD* = 40.70) |
| Political orientation  *very liberal* (0) to *very conservative* (100) | 35.40 (*SD* = 25.42) |
| Ethnicity | White/Caucasian: 1768 (76.0%)  African-American: 142 (6.1%)  Hispanic: 108 (4.6%)  Native American: 13 (0.6%)  Asian: 232 (10.0%)  Mixed: 54 (2.3%)  Other: 11 (0.5%) |
| Scientist or working in academia? | 220 (9.5%) |

*not recorded in Studies 1-3 and 6-7; *N* = 997
