# Supplementary material for: The Immoral Landscape? Scientists Are Associated with Violations of Morality
Source: PLoS One. 2016 Apr 5;11(4):e0152798. doi: 10.1371/journal.pone.0152798 (PMC4821584; doi:10.1371/journal.pone.0152798)
Supplement: S1 Scenarios — (DOCX) [file pone.0152798.s004.docx]

**S2 Scenarios**

**Study 1 and 4:**

**Please read the following description of Robert.**

When Robert was young, he began inflicting harm on animals. It started with just pulling the wings off flies, but eventually progressed to torturing stray cats and other animals in his neighborhood. As an adult, Robert found that he did not get much thrill from harming animals, so he began hurting people instead. He has killed 5 homeless people that he abducted from poor neighborhoods in his home city. Their dismembered bodies are currently buried in his basement.

**Study 2:**

**Please read the following description of Robert.**
Robert and his sister were traveling together in France. One night they were staying alone in a cabin near the beach. They decided that it would be interesting and fun if they tried making love. At very least it would be a new experience for each of them. Robert’s sister was already taking birth control pills, but Robert used a condom too, just to be safe. They both enjoyed it, but they decided not to do it again. They keep that night as a special secret between them, which makes them feel even closer to each other.

**Study 3, 5, and 10:**

**Please read the following description of Jack.**

On the way home from work, Jack decided to stop at the butcher shop to pick up something for dinner. He decided to roast a whole chicken. He got home, unwrapped the chicken carcass, and decided to make love to it. He used a condom, and fully sterilized the carcass when he was finished. He then roasted the chicken and ate it for dinner alongside a nice glass of Chardonnay.

**Study 6:**

**Please read the following description of Robert.**
Robert just moved into a new apartment. A coworker, Jan, helped Robert move his belongings. One day, Jan emailed Robert to see if Robert would help Jan move into a new apartment. Robert ignored the email and did not help Jan. Robert also enjoyed playing poker at a local bar. When he was playing with people he did not know well, Robert would usually cheat in order to win money.

**Study 7:**

**Please read the following description of Robert.**

Robert was on the bus on his way home from a long day at work. An overweight woman got on the bus. Rather than give up his seat for the woman, Robert made a number of cruel remarks to her about her appearance. After getting off the bus, Robert was walking to his apartment. A stray dog walked up to him to beg for food and Robert kicked it in the head, hard.

**Study 9:**

**Please read the following description of Jack.**

Jack's dog was killed by a car in front of his house. Jack had heard that dog meat was delicious, so he cut up the dog's body and cooked it and ate it for dinner.
